# Examining the joint contribution of maternal health, behavioral factors, psychosocial adversities, and environmental conditions during pregnancy for disparities in birth outcomes

**DOI:** 10.1186/s12884-026-09506-2

**Published:** 2026-06-19

**Authors:** Serim Lee, Allison A. Appleton

**Affiliations:** 1 Department of Social Welfare, Ewha Womans University, 52, Ewhayeodae-gil, Seodaemun-gu, Seoul, 03760 Republic of Korea; 2 College of Integrated Health Sciences, University at Albany, State University of New York, Albany, NY USA; 3 Department of Epidemiology and Biostatistics, College of Integrated Health Sciences, University at Albany, State University of New York, Albany, NY USA

**Keywords:** Maternal Health, Prenatal Exposures, Birth Outcomes, Gestational Age, Birth Size, Sociodemographic Disparities

## Abstract

**Background:**

Many pre-pregnancy and gestational exposures, such as maternal physical health, behavioral factors, psychosocial adversities, and environmental conditions during pregnancy, influence birth outcomes. These risk factors can co-occur, particularly among populations that experience health disparities, yet much of past researchers tend to examine them in isolation, which may lead inadvertently to some underestimation or mis-specification of risk. Therefore, this study examined the joint contribution of several maternal physical health, behaviors, psychosocial adversities, and environmental conditions during pregnancy on birth outcomes, and whether disparities were evident according to infant sex, maternal race/ethnicity, and education attainment.

**Methods:**

Participants were from the Albany Infant and Mother Study, a prospective observational cohort of socioeconomic and racial/ethnic diverse pregnant women and their infants (*n* = 291). Latent Class Analysis was employed to identify discrete groups with different levels and combinations of the following prenatal exposures: maternal health (pre-pregnancy body mass index, pregnancy conditions), behaviors (smoking, recreational drug use, western diet), psychosocial (depression, adverse childhood experiences, social support), and physical environment (lead exposure, indoor environmental contaminants). Linear regression analysis tested associations between exposure classes and birth outcomes.

**Results:**

Three latent classes were identified: Class 1 (47.7%; low-risk in all domains), Class 2 (42.9%; high-risk across all domains), and Class 3 (9.4%; high-risk in behavioral and environmental domains, but lower health and psychosocial risks). Compared to the low-risk referent Class 1, Class 2 was associated with lower birth weight, length, head circumference, more restricted fetal growth, and earlier gestational age at delivery (all *p* < 0.05). Class 3 infants also had marginally lower birth weight and increased intrauterine growth restriction (all *p* < 0.10). Associations were strongest among male infants, and participants from racial/ethnic and lower education backgrounds.

**Conclusions:**

This comprehensive examination of the combined effects of multiple exposures—encompassing physical health, behavioral, psychosocial and environmental domains— indicated that different patterns of multiple perinatal risks work in combination to influence birth outcomes and disparities at the earliest time in the life course. Research and practice focused on inequalities in maternal and perinatal health should consider the coordinated impacts of multiple risks simultaneously.

## Introduction

Gestation is a critical period of human development during which time adverse intrauterine exposures can influence developing body systems and risk of future health outcomes [1, 2]. Throughout pregnancy, the developing fetus undergoes significant growth, which can be negatively impacted by adversity in the womb and contribute to postnatal risk [3]. Preterm birth, intrauterine growth restriction, and small birth size can indicate the quality of the intrauterine environment [1], and are associated with increased risks of neonatal mortality, morbidity, developmental impairments, and chronic diseases later in life [3, 4]. Moreover, adverse birth outcomes are prevalent in the United States, and there are significant disparities, with lower socioeconomic and racial/ethnic groups being at higher risk compared to others [3]. Given the prevalence and disparities in birth outcomes, the marked neonatal vulnerability that follows and the enduring health risk over the life course, identifying the determinants of adverse birth outcomes is a critical public health concern.

Studies have shown that a wide range of individual-level maternal risk factors including pre-pregnancy and pregnancy morbidity, mental health, and behavioral factors [4], alongside social and environmental risks like psychosocial adversities and exposure to environmental toxicants can influence birth outcomes [5–11]. These risk factors can co-occur [12], yet perinatal researchers often examine pregnancy exposures in isolation. Doing so may lead inadvertently to some underestimation or mis-specification of risk for adverse birth outcomes [5]. The Integrated Socio-Environmental Model of Health and Well-Being (ISEM) is a framework that facilitates the investigation of the joint contribution of multiple exposures simultaneously, including during sensitive periods in the life course [13]. Following the ISEM, social and environmental factors may give rise to adverse experiences and shape health behaviors, which in turn may contribute to maternal perinatal health and subsequent birth outcomes. Some studies have investigated the joint contribution of prenatal social and environmental exposures in relation to offspring outcomes [5, 6, 9, 14]. For instance, in our prior work, we examined the joint contribution of prenatal lead and depression exposure for birth outcomes [5], and also the cumulative impact of prenatal exposure to positive neighborhood social and environmental conditions in relation to birth outcomes [6]. Both studies demonstrated enhanced precision of estimates when considering exposure synergies, though small sets of exposure combinations were considered. A more comprehensive analysis that incorporates a broader range of perinatal risk factors is warranted.

Prenatal exposures and their associations with birth outcomes are often patterned by infant sex, maternal race/ethnicity and socioeconomic factors. Racial and ethnic disparities in birth outcomes are well documented and are persistent [3]. For example, in 2022, preterm birth among Black women (14.6%) was approximately 50% higher than that among White (9.4%) and Hispanic women (10.1%) [3]. Additionally, maternal education is a significant predictor of birth outcomes, with lower levels associated with higher risks of preterm birth, small-for-gestational-age births, stillbirth, and neonatal and post-neonatal mortality [15]. In addition, prenatal exposure effects are often sex-specific, with male fetuses experiencing more risk than females [16, 17]. Therefore, it is important to identify the exposure patterns in the population that contribute to these disparities in order to develop interventions to promote equity for all.

Therefore, this study will investigate the joint contribution of maternal pre-pregnancy and perinatal morbidity, psychosocial adversity, behavioral factors, and environmental toxicant exposures in pregnancy on birth outcomes. To model this prenatal exposure complexity, we apply a Latent Class Analysis (LCA), a person-centered methodological approach that enables the identification of distinct patterns of multiple exposures in the underlying population, thereby overcoming traditional modeling approaches that examine exposures individually [18, 19]. We hypothesized that different patterns of perinatal exposures will be identified, and that these patterns will be associated with different thresholds of birth outcome risk. In addition, we examined whether associations between different classes (or combinations) of perinatal risks and birth outcomes varied according to infant sex, maternal race/ethnicity, and education. We follow the ISEM framework to provide a comprehensive examination of perinatal risks in association with birth outcome disparities.

## Methods

### Data

Participants were from the Albany Infant and Mother Study (AIMS), a prospective observational cohort of socioeconomic and racial/ethnic diverse pregnant women and their infants [5, 6, 20, 21]. Eligible participants were English-speaking women aged 18–40 with singleton pregnancies, recruited from an outpatient obstetrics clinic and enrolled around 27 weeks gestation. Study participation included a prenatal enrollment visit, and abstraction of clinical information from the participant’s medical record following the birth. During the prenatal enrollment visit, participants completed questionnaires covering demographics, pre-pregnancy health histories, and behaviors, psychosocial, and environmental factors during pregnancy. Toenail samples were collected at the pregnancy enrollment visit and assayed for metals exposure information. After delivery, clinical information on maternal health, delivery, and infant characteristics, including birth outcomes and infant sex, was obtained through a structured medical record review conducted by study physicians. Of the 300 mother-infant pairs enrolled, assessments were completed by 291. Institutional Review Board approval and informed consent were obtained from Albany Medical Center and the University at Albany, State University of New York, in accordance with research protocols.

### Measures

#### Birth outcomes

Birth size information was collected by delivery room personnel at the infant’s birth using standard procedures and instrumentation. Birth weight (g), birth length (cm), head circumference (cm) and gestational age at delivery (weeks) as recorded in the medical record were examined as study outcomes. In addition, we derived a cephalization index (head circumference (cm)/birth weight (g)), indicating the degree of asymmetry in intrauterine fetal growth [22, 23]. Cephalization index was logarithmically transformed (base 10) due to a skewed distribution. All birth outcome variables were measured once at delivery.

#### Maternal characteristics

##### Physical health

Maternal self-reported pre-pregnancy height and weight was used to derive pre-pregnancy body mass index (BMI; weight kg/height m^2^), which was then categorized based on CDC standards [24]: underweight (< 18.5), normal weight (18.5–24.9), overweight (25-29.9), and obese (30+). A dichotomous variable indicating overweight or obesity versus normal and underweight was derived.

A pregnancy conditions variable was derived to allow for control of the full range of complications that could arise in pregnancy (both in terms of severity and impact on outcomes). Pregnancy conditions were abstracted from the medical record and included pregnancy morbidity (diagnoses of gestational diabetes, preeclampsia, eclampsia), placental abnormalities (abruption, previa, accreta, marginal bleed), gynecologic bacterial infections (Group B Streptococcus, Chorioamnionitis), and preterm premature rupture of membranes. A dichotomous variable indicating whether or not participants had any one of these conditions was derived. Individual conditions were not examined due to low prevalence of each condition in the study population (e.g., < 10%).

##### Behaviors during pregnancy

Smoking, recreational drug use, and diet during pregnancy were assessed at the prenatal enrollment visit via self-report. Smoking during pregnancy was assessed with one question “Do you currently smoke?” (yes/no). For recreational drug use, participants indicated whether or not they had used any of the following substances during the focal pregnancy: marijuana, heroin, methadone, cocaine, crack, methamphetamine, LSD, PCP, mushrooms, recreational prescription drugs, or other recreational drugs. Participants were classified as using recreational drugs during pregnancy if they indicated “yes” to using at least one drug type. To assess diet in pregnancy, participants completed an abbreviated Food Frequency Questionnaire (FFQ) [25, 26], which assessed the frequency of consumption of various food types. A western dietary score was derived, indicating the frequency of food consumption across Western categories, such as whole milk, ice cream, butter, processed meats, hot dogs/hamburgers, beef/pork/lamb, white bread, bakery items, pasta, potatoes, and soft drinks. A sum score was derived, with higher scores indicating greater adherence to a western style diet. This score was then dichotomized with the top tertile of the distribution indicating high adherence to western diet style.

##### Psychosocial adversity

Maternal history of adverse childhood experiences, as well as depression and social support during pregnancy were assessed via self-report at the prenatal enrollment visit. Depression was measured with the Edinburgh Postnatal Depression Scale (EPDS; α = 0.87) which has been validated for use among perinatal populations because it focuses on the cognitive and affective features of depression rather than somatic complaints that are common in pregnancy and the postpartum period [27]. A dichotomous variable indicating clinically significant depressive symptoms (scores > 12.5) was examined in analysis. Adverse Childhood Experiences (ACEs) were assessed using the ACEs questionnaire [28], a 10-item retrospective account of adversities occurring before age 18 and included neglect (emotional, physical), abuse (emotional, physical, sexual), household dysfunction (witnessing domestic violence, having a household member imprisoned, experiencing household member mental illness, and drug/alcohol abuse in the home), and parental separation/divorce. Sum scores were derived indicating the total number of ACEs, and then a dichotomous variable was created to distinguish between high (*≥* 4) and lower (< 4) ACEs scores. Social support during pregnancy was measured using the 12-item Interpersonal Support Evaluation List (ISEL; α = 0.86) [29], which assessed perceived availability of social resources and relationships in terms of belongingness and tangible supports. Total scores were calculated, and a dichotomous variable was derived to indicate low and high social support. As there are no standard cutpoint to signify high or low social support, we used the bottom tertile of the distribution to signify low social support.

##### Environmental factors

Exposure to the metal lead was assessed from maternal toenail clippings and indoor environmental contaminants exposures were assessed via questionnaire, both collected at the prenatal enrollment visit. Lead exposure assessment has been described elsewhere, including QA and QC procedures carried out by the laboratory [5]. Briefly, maternal toenail clippings collected and stored at room temperature. Prior to assay, nails were washed to remove external contamination, dried, and subjected to microwave digestion. Samples were analyzed with inductively coupled plasma mass spectrometry with Element2 high-resolution ICP-MS (Thermon Finnegan, Bremen, Germany) and a quadrupole collision cell 7500c ORS ICP-MS (Agilent, Santa, Clara, CA, USA). All sample preparations and analyses were carried out in a trace-metal clean HEPA-filtered-air environment. Lead concentrations (range: 0.02 to 12.67 µg/g) were dichotomized with the top tertile of the distribution indicating higher levels of exposure (range: 0.71 to 12.67 µg/g). Indoor environmental exposures were assessed with a 14-item survey [30] that assessed exposures and quality of the home environment such as the age of the home, noise, presence of mold, pests (e.g., mice, rats, cockroaches), frequency of indoor pesticide application and the frequency of use of chemical household and personal care products. A cumulative sum score indicating the total number of indoor home physical environment exposures was derived, with higher scores indicating greater exposure to indoor environmental risks. This score was dichotomized using the top tertile of the distribution indicating a high degree of indoor environmental contaminant exposure.

#### Covariates

Self-reported maternal age in years, race/ethnicity (dichotomized as White and not Hispanic versus Hispanic and/or a racial/ethnic minority (Black or African American/Asian or Pacific Islander/American Indian or Alaskan Native)), and education attainment (dichotomized as high school/GED or less versus more than high school) were collected at the enrollment visit. Infant sex (male/female) was abstracted from medical records. These variables were included as covariates in the BCH models and subsequent regression analyses to account for potential confounding.

### Analysis

First, to address missing data, we utilized a full information maximum likelihood (FIML) approach implemented in Mplus version 8.9 [31]. This method has been extensively validated through simulation studies, affirming its efficacy in minimizing bias related to missing data [32, 33], including in LCA [34]. Missingness across key study variables had an average rate of 6.8% (Standard Deviation [SD] = 8.4%). Then, we calculated descriptive statistics for the full sample, and bivariate associations were examined for each study variable with infant sex, race/ethnicity, and education attainment with independent *t* and χ^2^ tests. Next, we conducted a LCA to uncover distinct patterns of maternal perinatal risks. LCA, a statistical method aimed at identifying latent classes or unobserved groups based on categorical observed variables [18], adopts a person-centered approach to identify similar patterns among respondents across multiple indicators [33]. LCA model fit was assessed using the Akaike information criterion (AIC), Bayesian information criterion (BIC), and sample-size adjusted Bayesian information criterion, with lower values indicating better fit [35]. Additionally, the Lo–Mendell–Rubin adjusted likelihood ratio test (LMR-LRT) and bootstrap likelihood ratio test (BLRT) were employed to determine whether our specified model provided a superior fit compared to a model with one less class (K-1) [35]. Classification accuracy was evaluated through entropy values, with higher scores indicating clearer classification and stronger distinction among classes [35].

To assess equality across latent classes, we employed the Bolck, Croon, and Hagenaars (BCH) method, incorporating auxiliary variables to examine class-specific relationships with external variables [36]. These variables included measures of birth outcomes and covariates. Finally, we examined the association between the latent classes of maternal perinatal risks with birth outcomes via linear regression. These models were then stratified by infant sex, maternal race/ethnicity, and education attainment to examine possible disparities in the latent class and birth outcome associations. The VIF values for all models were all below 10, indicating the absence of multicollinearity for all models [37]. Analyses were conducted using Mplus version 8.9 (Muthén & Muthén, 1998–2004) and SPSS version 29.

## Results

Table 1 presents a descriptive analysis of birth outcomes, maternal characteristics, and covariates, stratified according to the infant’s sex, maternal race/ethnicity, and education attainment. On average, participants were 28.46 years old (SD = 5.50), with 59.8% identifying as White not-Hispanic, and 38.1% reporting that their highest level of education attained was high school or less. On average, birth weight was 3,242.67 g (SD = 634.78), birth length was 48.68 cm (SD = 3.32), head circumference was 33.43 cm (SD = 3.32), cephalization index was 0.05 cm/g (SD = 0.18), and gestational age at delivery was 38.81 weeks (SD = 2.12).

**Table 1:** Participant characteristics

	Total (*n* = 291)	Male Infants (*n* = 155)	Female Infants (*n* = 136)	*P*	White, not Hispanic (*n* = 174)	Hispanic and/or racial/ethnic minority (*n* = 117)	*p*	High Education (*n* = 111)	Low Education (*n* = 180)	*p*
c	*n*(%)/M(SD)	*n*(%)/M(SD)	*n*(%)/M(SD)		*n*(%)/M(SD)	*n*(%)/M(SD)		*n*(%)/M(SD)	*n*(%)/M(SD)	
Infant characteristics										
Birth weight (g)	3242.67 (634.78)	3289.99 (665.07)	3195.35 (601.69)	0.22	3350.50 (579.25)	3081.40 (681.18)	0.001	3283.26 (588.24)	3172.85 (705.36)	0.16
Birth length (cm)	48.68 (3.32)	48.83 (3.47)	48.52 (3.17)	0.45	49.23 (2.98)	47.85 (3.64)	0.001	48.96 (3.04)	48.19 (3.73)	0.06
Head circumference (cm)	33.43 (2.18)	33.52 (2.26)	33.34 (2.10)	0.51	33.80 (2.16)	32.87 (2.11)	0.001	33.68 (1.95)	32.98 (2.49)	0.01
Birth cephalization index (cm/g)	0.05 (0.18)	0.04 (0.20)	0.05 (0.17)	0.67	0.02 (0.02)	0.08 (0.20)	0.008	0.04 (0.17)	0.05 (0.20)	0.74
Gestational age at birth, weeks	38.81 (2.12)	38.74 (2.16)	38.89 (2.09)	0.54	39.02 (1.54)	38.51 (2.76)	0.08	38.96 (1.90)	38.57 (2.44)	0.14
Infants sex, male	155 (53.3%)	-	-	-	91 (52.3%)	64 (54.7%)	0.72	92 (51.1%)	63 (56.8%)	0.39
Infant sex, female	136 (46.7%)	-	-	-	83 (47.7%)	53 (45.3%)		88 (48.9%)	48 (43.2%)	
Maternal characteristics										
Age	28.46 (5.50)	28.53 (5.53)	28.48 (5.49)	0.81	28.83 (5.42)	27.91 (5.60)	0.16	30.07 (5.09)	25.85 (5.15)	0.000
Race, White not Hispanic	174 (59.8%)	91 (58.7%)	83 (61.0%)	0.72	-	-	-	114 (63.3%)	60 (54.1%)	0.14
Race, Hispanic and/or racial/ethnic minority	117 (40.2%)	64 (41.3%)	53 (39.0%)		-	-	-	66 (36.7%)	51 (45.9%)	
Education, *≤* high school/GED	111 (38.1%)	63 (40.6%)	48 (35.3%)	0.39	60 (34.5%)	51 (43.6%)	0.14	-	-	-
Education, > high school/GED	180 (61.9%)	92 (59.4%)	88 (64.7%)		114 (65.5%)	66 (56.4%)		-	-	-
Pregnancy overweight or obesity, yes	168 (57.7%)	94 (60.6%)	74 (54.4%)	0.56	99 (56.9%)	69 (59.0%)	0.80	103 (57.2%)	65 (58.6%)	0.54
Pregnancy condition, yes	107 (36.8%)	46 (29.7%)	61 (44.9%)	0.01	58 (33.3%)	49 (41.9%)	0.17	70 (38.9%)	37 (33.3%)	0.38
Smoked during pregnancy, yes	44 (15.1%)	30 (19.4%)	14 (10.3%)	0.03	32 (18.4%)	12 (10.3%)	0.06	17 (9.4%)	27 (24.3%)	0.001
Recreational drug use during pregnancy, yes	61 (21.0%)	34 (21.9%)	27 (19.9%)	0.77	37 (21.3%)	24 (20.5%)	0.87	36 (20.0%)	25 (22.5%)	0.65
Western diet, yes	90 (30.9%)	55 (35.5%)	35 (25.7%)	0.19	51 (29.3%)	39 (33.3%)	0.11	47 (26.1%)	43 (38.7%)	0.07
Depression, yes	64 (22.0%)	38 (24.5%)	26 (19.1%)	0.12	31 (17.8%)	33 (28.2%)	0.11	37 (20.6%)	27 (24.3%)	0.74
Adverse childhood experiences, yes	89 (30.6%)	51 (32.9%)	38 (27.9%)	0.58	56 (32.2%)	33 (28.2%)	0.36	53 (29.4%)	36 (32.4%)	0.25
Low social support, yes	180 (61.9%)	99 (63.9%)	81 (59.6%)	0.30	95 (54.6%)	85 (72.6%)	0.007	105 (58.3%)	75 (67.6%)	0.27
Lead exposure, high	93 (32.0%)	60 (38.7%)	33 (24.4%)	0.02	59 (33.9%)	34 (29.1%)	0.12	45 (25.0%)	48 (43.2%)	0.000
Indoor environmental risks, high	80 (27.5%)	43 (27.7%)	37 (27.2%)	0.46	50 (28.7%)	30 (25.6%)	0.72	52 (28.9%)	28 (25.2%)	0.77

Note ^p-value for independent t or χ2 tests for continuous and categorical variables respectively

Regarding maternal characteristics, 57.5% were classified as overweight or obese prior to pregnancy, and 36.8% reported at least one pregnancy condition. The prevalence of pregnancy conditions varied across conditions, including gestational diabetes (7.6%), gestational hypertension (7.2%), preeclampsia (10.7%), eclampsia (7.6%), placental abnormalities (abruption: 1.7%, previa: 1.0%, accreta: 6.9%, marginal bleed: 0.7%), gynecologic bacterial infections (Group B Streptococcus: 23.7%, chorioamnionitis: 9.3%), and preterm premature rupture of membranes (2.4%). Additionally, 15.1% reported smoking during pregnancy, while 21.0% reported recreational drug use during pregnancy. A total of 30.9% exhibited Western dietary patterns. Mental health factors indicated that 22.0% experienced depression, and 30.6% reported 4 or more ACEs. Furthermore, 61.9% reported low social support. Approximately one-third of participants had high lead and high degrees of indoor environmental risks, which corresponds to the tertiles used for dichotomizing these variables.

Pregnancy complications were more prevalent among female infants compared to males (29.7% for males vs. 44.9% for females; *p* < 0.05). Racial/ethnic minority participants had significantly higher rates of low social support compared to non-Hispanic White participants (54.6% for non-Hispanic White vs. 72.6% for racial/ethnic minority; *p* < 0.01). Racial/ethnic minority infants had significantly lower birth weight, length, head circumference, and more restricted fetal growth (all *p* < 0.01). Participants with low education were significantly younger (Mean [M] = 30.07 for high education vs. M = 25.85 for low education; *p* < 0.001), had a higher rate of smoking (9.4% for high education vs. 24.3% for low education; *p* < 0.01), and a higher degree of lead exposure (25.0% for high education vs. 43.2% for low education; *p* < 0.001).

**Fig. 1:**
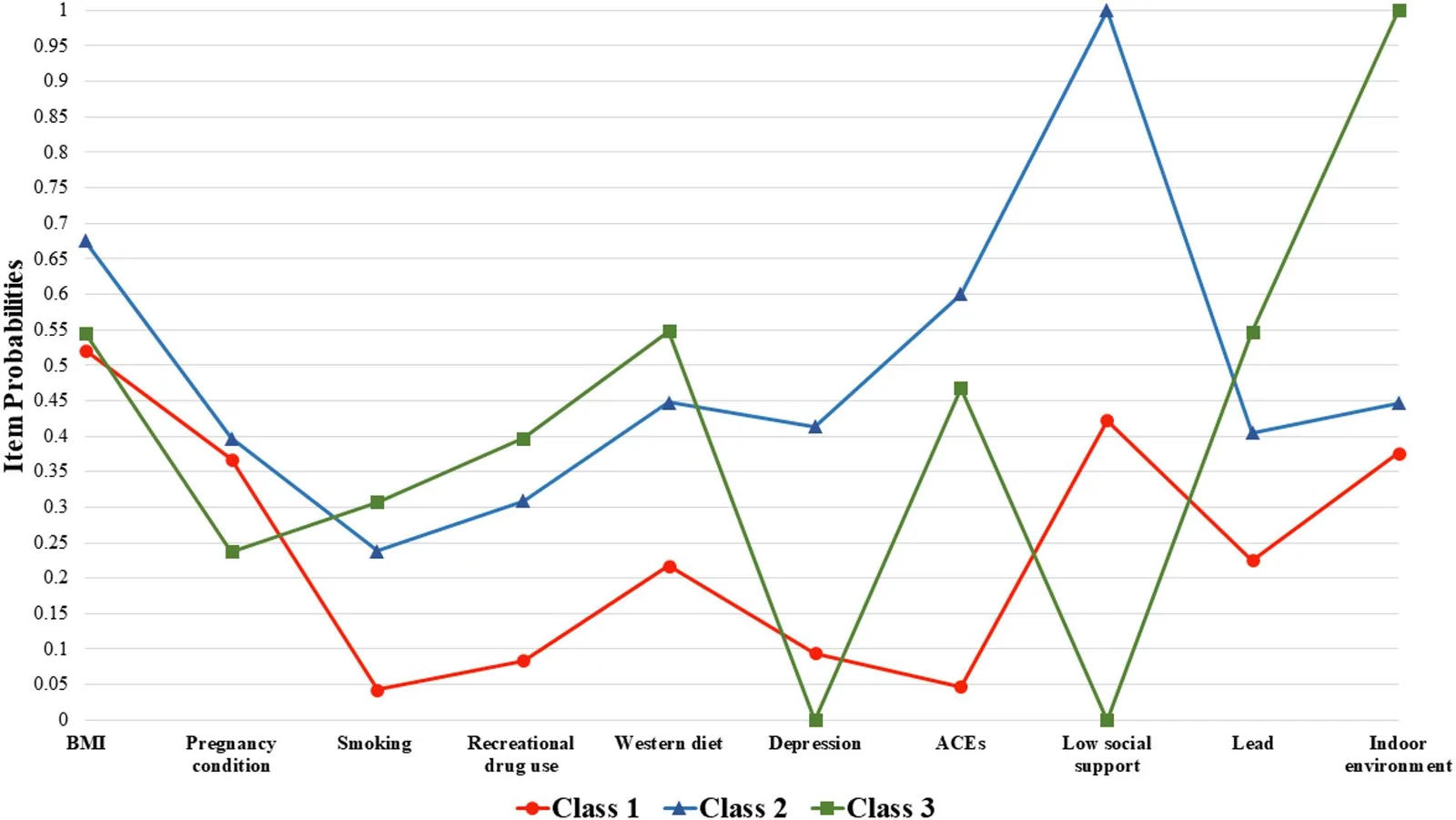
Latent classes and item probabilities for maternal characteristics

The LCA identified a three-class model as the most appropriate fit for the maternal characteristics data (see Fig. 1; Table 2). Class 1 (47.7%) emerged as a low-risk group as it exhibited among the lowest levels of all maternal health, behavior, psychosocial, and environmental risks.

**Table 2:** Fit indices for latent class analysis

Number of Latent Class	AIC	BIC	Adjusted BIC	LMR_LRT (*p*-value)	BLRT (*p*-value)	Entropy
1-class	3304.00	3340.73	3309.02	-	-	-
2-class	3242.95	3320.09	3253.50	0.001	0.00	0.52
3-class	3238.45	3355.99	3254.51	0.02	0.23	0.70
4-class	3237.60	3395.55	3259.19	0.44	0.30	0.65

*Abbreviations*: *AIC* Akaike Information Criterion, *BIC* Bayesian Information Criterion, *LMR_LRT* Lo-Mendell-Rubin adjusted Likelihood Ratio Test, *BLRT* Bootstrap Likelihood Ratio Test

**Fig. 2:**
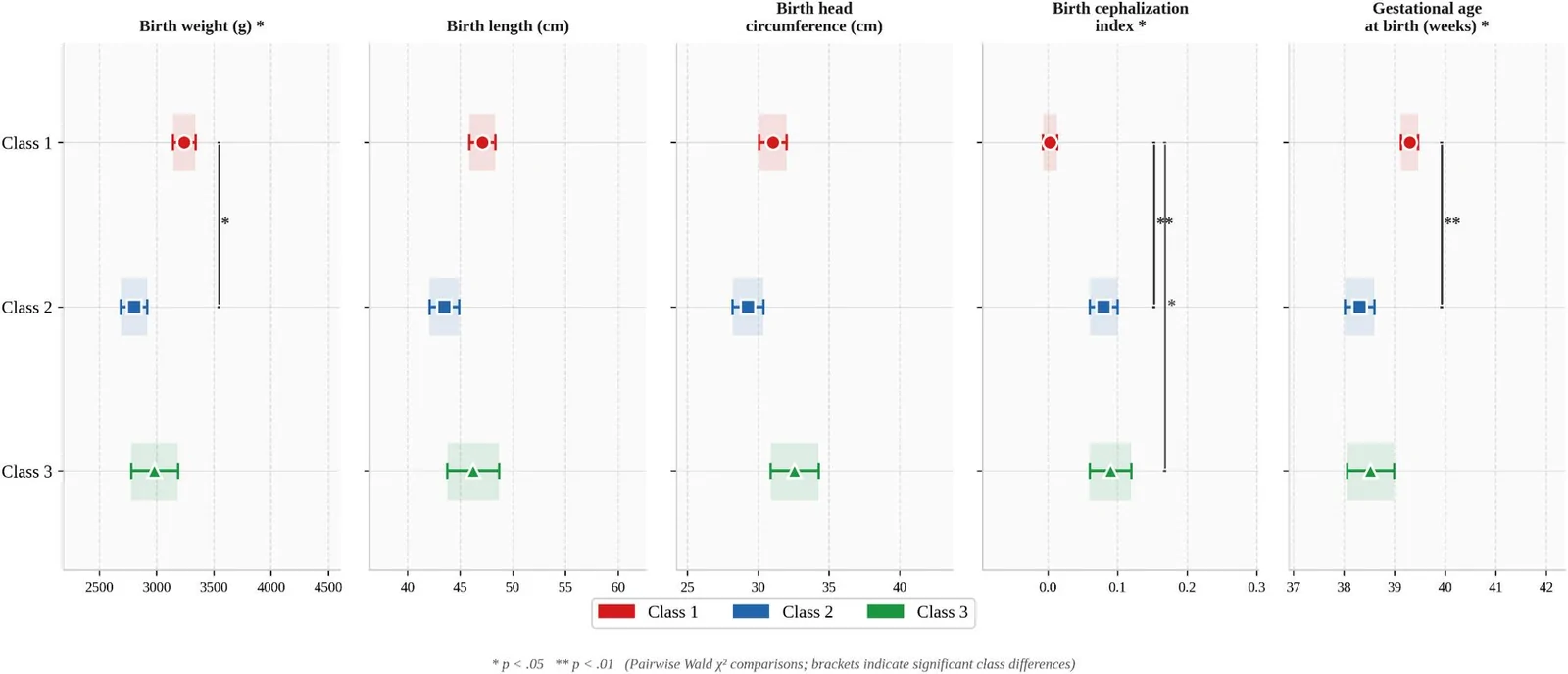
Distribution of birth outcomes across latent classes derived from BCH analysis (Mean ± SE)

Class 2 (42.9%) emerged as a high risk group as it displayed among the highest levels across all indicators of maternal health (BMI, pregnancy conditions), behavior (smoking, recreational drug use, western diet), psychosocial (depression, ACEs, low social support) and environmental risks (lead, indoor exposures). Class 3 (9.4%) emerged as a second high risk group that had the highest levels of behavioral and environmental risks, but lower levels of psychosocial and physical health risks. Table 3 presents equality across classes according to birth outcomes and study covariates using the BCH procedure. It demonstrates that Class 1 infants had significantly higher birth weight compared to Class 2 infants (χ² = 6.675, *p* < 0.05). Regarding the birth cephalization index, Class 1 exhibits a significantly lower index compared to both Class 2 (χ² = 6.917, *p* < 0.01) and Class 3 (χ² = 4.012, *p* < 0.05). Additionally, Class 1 shows significantly higher gestational age at birth in weeks compared to Class 3 (χ² = 6.833, *p* < 0.01) (See Fig. 2).

**Table 3:** Bivariate associations and equality tests across classes

		*M*	*SE*	$\:{x}^{2}$	Contrast	$\:{x}^{2}$
Maternal age	Class 1	29.20	0.56	3.47	Class 1 vs. 2	3.26
	Class 2	27.57	0.57		Class 1 vs. 3	0.10
	Class 3	28.65	1.48		Class 2 vs. 3	0.47
Race: Hispanic and/or racial/ethnic minority	Class 1	0.35	0.05	3.07	Class 1 vs. 2	1.99
	Class 2	0.47	0.05		Class 1 vs. 3	0.10
	Class 3	0.31	0.11		Class 2 vs. 3	1.62
Education: High school/GED or less	Class 1	0.28	0.04	5.87	Class 1 vs. 2	5.68*
	Class 2	0.47	0.05		Class 1 vs. 3	1.84
	Class 3	0.47	0.12		Class 2 vs. 3	0.001
Infants’ sex: Male	Class 1	0.45	0.05	3.85	Class 1 vs. 2	3.74
	Class 2	0.61	0.05		Class 1 vs. 3	0.28
	Class 3	0.53	0.12		Class 2 vs. 3	0.37
Birth weight, g	Class 1	3243.24	98.65	6.68*	Class 1 vs. 2	6.67*
	Class 2	2804.95	114.70		Class 1 vs. 3	1.12
	Class 3	2983.83	203.18		Class 2 vs. 3	0.60
Birth length, cm	Class 1	47.13	1.22	3.20	Class 1 vs. 2	2.94
	Class 2	43.51	1.42		Class 1 vs. 3	0.08
	Class 3	46.25	2.46		Class 2 vs. 3	0.95
Birth head circumference, cm	Class 1	31.05	0.98	3.08	Class 1 vs. 2	1.13
	Class 2	29.28	1.09		Class 1 vs. 3	0.50
	Class 3	32.58	1.70		Class 2 vs. 3	2.75
Birth cephalization index	Class 1	0.003	0.01	8.04*	Class 1 vs. 2	6.91**
	Class 2	0.08	0.02		Class 1 vs. 3	4.01*
	Class 3	0.09	0.03		Class 2 vs. 3	0.01
Gestational age at birth, weeks	Class 1	39.30	0.17	7.20*	Class 1 vs. 2	6.83**
	Class 2	38.31	0.29		Class 1 vs. 3	2.08
	Class 3	38.53	0.46		Class 2 vs. 3	0.17

* *p* < 0.05; **p < 0.01; ***p < 0.001

Tables 4 and 5 display the multivariable linear regression associations for latent classes of perinatal risk with infant outcomes for the full sample, and also stratified by infant sex, maternal race/ethnicity and education attainment. In the full sample, compared to Class 1 (low-risk in all domains), Class 2 (high-risk across all domains) showed significant associations with all birth outcomes. Specifically, compared to Class 1, infants in Class 2 had significantly lower birth weight (regression coefficient [B] = -240.23, standard error [SE] = 79.64, *p* < 0.01), shorter birth length (B = -1.45, SE = 0.42, *p* < 0.01), smaller head circumference (B = -0.59, SE = 0.28, *p* < 0.05), a higher birth cephalization index (B = 0.06, SE = 0.02, *p* < 0.05), and earlier gestational age at birth (B = -0.63, SE = 0.27, *p* < 0.05). In the full sample, compared to Class 1, Class 3 (high-risk in behavioral and environmental domains, but lower health and psychosocial risks) infants had marginally lower birth weight (B = -228.33, SE = 136.21, *p* < 0.10) and increased cephalization index (B = 0.07, SE = 0.04, *p* = 0.10).

**Table 4:** Multivariable linear regression associations for latent classes of perinatal risk with infant outcomes among Albany infant and mother study participants, and also stratified by infant sex

Variables (Ref)	Total		Male		Female	
	B(SE)	*p*-value	B(SE)	*p*-value	B(SE)	*p*-value
Birth weight						
Age	3.29 (7.34)	0.65	2.82 (11.50)	0.80	4.76 (9.73)	0.62
Race (White/Not Hispanic)	-247.54 (76.92)	0.001	-209.36 (116.28)	0.07	-257.39 (105.67)	0.01
Education (high)	-35.16 (84.25)	0.68	-5.31 (131.91)	0.96	-102.05 (115.29)	0.37
Infants’ sex (female)	124.41 (74.91)	0.09	-	-	-	-
Class 2	-240.23 (79.64)	0.003	-318.71 (117.26)	0.007	-137.44 (110.91)	0.21
Class 3	-228.33 (136.21)	0.09	-149.73 (214.83)	0.48	-312.22 (183.63)	0.09
Class 1 (ref)	-	-	-	-	-	-
*F*(*df*)	4.29 (6)	0.00	2.35 (5)	0.04	2.98 (5)	0.01
$\:{R}^{2}$	0.09		0.08		0.10	
Birth length						
Age	-0.001 (0.04)	0.97	0.010 (0.06)	0.87	-0.02 (0.05)	0.74
Race (White, Not Hispanic)	-1.22 (0.40)	0.003	-1.15 (0.61)	0.06	-1.08 (0.54)	0.04
Education (high)	-0.43 (0.44)	0.32	-0.03 (0.70)	0.96	-0.99 (0.59)	0.09
Infants’ sex (female)	0.49 (0.39)	0.21	-	-	-	-
Class 2	-1.45 (0.42)	0.001	-1.25 (0.62)	0.04	-1.59 (0.57)	0.006
Class 3	-1.14 (0.71)	0.11	-0.45 (1.13)	0.69	-2.03 (0.94)	0.03
Class 1 (ref)	-	-	-	-	-	-
*F*(*df*)	4.64 (6)	0.00	1.72 (5)	0.13	4.57 (5)	0.001
$\:{R}^{2}$	0.10		0.06		0.15	
Birth head circumference						
Age	0.06 (0.03)	0.02	0.05 (0.04)	0.22	0.06 (0.03)	0.07
Race (White, Not Hispanic)	-0.78 (0.27)	0.004	-1.20 (0.39)	0.003	-0.26 (0.37)	0.49
Education (high)	-0.29 (0.29)	0.32	-0.14 (0.45)	0.74	-0.64 (0.41)	0.12
Infants’ sex (female)	0.25 (0.26)	0.34	-	-	-	-
Class 2	-0.59 (0.28)	0.03	-0.51 (0.40)	0.19	-0.58 (0.39)	0.14
Class 3	-0.09 (0.47)	0.84	0.16 (0.72)	0.82	-0.62 (0.64)	0.33
Class 1 (ref)	-	-	-	-	-	-
*F*(*df*)	4.73 (6)	0.00	3.33 (5)	0.007	3.13 (5)	0.01
$\:{R}^{2}$	0.10		0.12		0.11	
Birth cephalization index						
Age	-7.362E-5 (0.002)	0.97	-0.001 (0.003)	0.72	0.001 (0.003)	0.83
Race (White, Not Hispanic)	0.006 (0.02)	0.01	0.05(0.04)	0.19	0.06 (0.03)	0.04
Education (high)	-0.01 (0.03)	0.74	-0.02(0.04)	0.68	0.01 (0.03)	0.79
Infants’ sex (female)	-0.02 (0.02)	0.44				
Class 2	0.06 (0.02)	0.01	0.09 (0.04)	0.01	0.02 (0.03)	0.47
Class 3	0.07 (0.04)	0.09	0.05 (0.07)	0.41	0.08 (0.05)	0.10
Class 1 (ref)	-	-	-	-	-	-
*F*(*df*)	2.45 (6)	0.02	1.76 (5)	0.12	1.68 (5)	0.14
$\:{R}^{2}$	0.05		0.07		0.06	
Gestational age at birth in weeks						
Age	0.02 (0.03)	0.42	0.05 (0.04)	0.18	-0.004 (0.04)	0.90
Race (White, Not Hispanic)	-0.42 (0.26)	0.11	-0.53 (0.38)	0.16	-0.24 (0.38)	0.53
Education (high)	-0.16 (0.29)	0.58	0.07 (0.43)	0.88	-0.41 (0.42)	0.32
Infants’ sex (female)	-0.08 (0.26)	0.74				
Class 2	-0.63 (0.27)	0.02	-0.86 (0.39)	0.02	-0.42 (0.40)	0.29
Class 3	-0.47 (0.47)	0.31	-0.60 (0.70)	0.39	-0.62 (0.66)	0.35
Class 1 (ref)	-	-	-	-	-	-
*F*(*df*)	1.98 (6)	0.06	1.93 (5)	0.09	0.80 (5)	0.54
$\:{R}^{2}$	0.04		0.07		0.03	

**Table 5:** Multivariable linear regression associations for latent classes of perinatal risk according to maternal race/ethnicity and education attainment

Variables (Ref)	White, not Hispanic		Hispanic and/or racial/ethnic minority		High education		Low education	
	B(SE)	*p*-value	B(SE)	*p*-value	B(SE)	*p*-value	B(SE)	*p*-value
Birth weight								
Age	-1.96 (8.91)	0.82	11.68 (12.78)	0.36	12.97 (8.75)	0.14	-6.69 (13.32)	0.61
Race (White, not Hispanic)					-317.54 (91.80)	0.001	-167.09 (138.47)	0.23
Education (high)	-125.22 (105.98)	0.23	90.54 (143.91)	0.53				
Infants’ sex (female)	115.63 (91.85)	0.21	165.50 (133.40)	0.21	62.72 (89.63)	0.48	181.97 (138.56)	0.19
Class 2	-201.34 (99.06)	0.04	-277.42 (134.45)	0.04	-73.78 (95.23)	0.44	-454.78 (143.78)	0.002
Class 3	-203.85 (155.43)	0.19	-191.53 (278.50)	0.49	-150.59 (163.92)	0.36	-326.61 (249.66)	0.19
Class 1 (ref)	-	-	-	-	-	-	-	-
*F*(*df*)	1.62(5)	0.15	1.33 (5)	0.25	3.51 (5)	0.005	2.77 (5)	0.02
$\:{R}^{2}$	0.05		0.06		0.10		0.13	
Birth length								
Age	-0.07 (0.05)	0.12	0.11 (0.07)	0.12	0.04 (0.05)	0.36	- 0.05 (0.07)	0.51
Race (White, not Hispanic)					-1.51 (0.47)	0.002	- 0.77 (0.74)	0.30
Education (high)	-1.07 (0.54)	0.04	0.48 (0.76)	0.53				
Infants’ sex (female)	0.56 (0.47)	0.23	0.62 (0.70)	0.38	0.06 (0.46)	0.89	1.06 (0.74)	0.15
Class 2	-1.20 (0.50)	0.01	-1.71 (0.71)	0.01	- 0.91 (0.49)	0.06	-2.01 (0.77)	0.01
Class 3	-1.08 (0.79)	0.17	- 0.75 (1.47)	0.61	- 0.98 (0.85)	0.24	-1.32 (1.34)	0.32
Class 1 (ref)	-	-	-	-	-	-	-	-
*F*(*df*)	2.61 (5)	0.02	1.81 (5)	0.11	3.59 (5)	0.004	2.18 (5)	0.06
$\:{R}^{2}$	0.08		0.08		0.10		0.10	
Birth head circumference								
Age	0.07 (0.03)	0.04	0.04 (0.04)	0.29	0.08 (0.03)	0.004	0.03 (0.05)	0.49
Race (White, not Hispanic)					-1.14 (0.30)	0.00	- 0.27 (0.51)	0.60
Education (high)	-0.64 (0.39)	0.10	0.08 (0.46)	0.86				
Infants’ sex (female)	0.61 (0.34)	0.07	- 0.22 (0.43)	0.60	0.002 (0.29)	0.99	0.53 (0.51)	0.31
Class 2	-0.55 (0.36)	0.13	- 0.57 (0.43)	0.18	-0.07 (0.31)	0.81	-1.19 (0.54)	0.02
Class 3	-0.17 (0.56)	0.75	0.01 (0.88)	0.98	0.03 (0.53)	0.96	- 0.18 (0.92)	0.84
Class 1 (ref)	-	-	-	-	-	-	-	-
*F*(*df*)	3.49 (5)	0.005	0.73 (5)	0.60	4.99 (5)	0.00	1.45 (5)	0.21
$\:{R}^{2}$	0.10		0.04		0.13		0.07	
Birth cephalization index								
Age	0.003 (0.003)	0.21	- 0.01(0.004)	0.18	- 0.003 (0.003)	0.26	0.004 (0.004)	0.36
Race (White, not Hispanic)					0.07 (0.03)	0.01	0.04 (0.04)	0.31
Education (high)	0.02 (0.003)	0.52	- 0.05 (0.04)	0.25				
Infants’ sex (female)	- 0.02 (0.03)	0.54	- 0.03 (0.04)	0.53	- 0.01 (0.03)	0.79	- 0.02 (0.04)	0.60
Class 2	0.06 (0.03)	0.06	0.06 (0.04)	0.14	0.03 (0.03)	0.32	0.09 (0.04)	0.03
Class 3	0.06 (0.05)	0.20	0.06 (0.08)	0.44	0.05 (0.05)	0.30	0.07 (0.07)	0.31
Class 1 (ref)	-	-	-	-	-	-	-	-
*F*(*df*)	1.22 (5)	0.30	1.02 (5)	0.40	2.32 (5)	0.04	1.44 (5)	0.21
$\:{R}^{2}$	0.04		0.05		0.07		0.07	
Gestational age at birth in weeks								
Age	0.00 (0.02)	0.98	0.05 (0.05)	0.35	0.04 (0.03)	0.18	- 0.003 (0.05)	0.94
Race (White, not Hispanic)					- 0.53 (0.31)	0.08	- 0.28 (0.50)	0.57
Education (high)	-0.42 (0.29)	0.14	0.06 (0.59)	0.91				
Infants’ sex (female)	0.03 (0.25)	0.89	-0.25 (0.55)	0.65	-0.22 (0.30)	0.47	- 0.05 (0.50)	0.91
Class 2	- 0.27 (0.27)	0.30	-1.09 (0.55)	0.05	- 0.25 (0.32)	0.43	-1.11 (0.52)	0.03
Class 3	- 0.23 (0.42)	0.57	-0.90 (1.13)	0.43	-0.54 (0.55)	0.32	- 0.33 (0.90)	0.71
Class 1 (ref)	-	-	-	-	-	-	-	-
*F*(*df*)	0.89 (5)	0.48	1.10 (5)	0.36	1.59 (5)	0.16	1.01 (5)	0.41
$\:{R}^{2}$	0.03		0.05		0.05		0.05	

When stratifying by infant sex, compared to Class 1, Class 2 male infants had significantly lower birth weight (B = -318.71, SE = 117.26, *p* < 0.01), shorter birth length (B = -1.25, SE = 0.62, *p* < 0.05), higher birth cephalization index (B = 0.09, SE = 0.04, *p* < 0.05), and earlier gestational age at birth (B = -0.86, SE = 0.39, *p* < 0.05). For female infants, both Class 2 and Class 3 were associated with shorter birth length (B = -1.59, SE = 0.57, *p* < 0.01 for Class 2, and B = -2.03, SE = 0.94, *p* < 0.05 for Class 3). Class 3 female infants also exhibited lower birth weight (B = -312.22, SE = 183.63, *p* = 0.10).

When stratifying by maternal race/ethnicity, compared to Class 1, Class 2 infants of non-Hispanic White mothers had significantly lower birth weight (B = -201.34, SE = 99.06, *p* < 0.05) and shorter birth length (B = -1.20, SE = 0.50, *p* < 0.05). Similarly, Class 2 infants of racial/ethnic minority mothers had significantly lower birth weight (B = -277.42, SE = 134.45, *p* < 0.05) and shorter birth length (B = -1.71, SE = 0.71, *p* < 0.05). Class 3 was not associated with any outcome in race/ethnicity stratified models.

When stratifying by maternal education, compared to Class 1, Class 2 infants of mothers with low education had lower birth weight (B = -454.78, SE = 143.78, *p* < 0.01), shorter birth length (B = -2.01, SE = 0.77, *p* < 0.05), smaller head circumference (B = -1.19, SE = 0.54, *p* < 0.05), higher birth cephalization index (B = 0.09, SE = 0.04, *p* < 0.05), and earlier gestational age at birth (B = -1.11, SE = 0.52, *p* < 0.05). Class 3 was not associated with any outcome in education stratified models.

## Discussion

The present study investigated the joint contribution of multiple pre-pregnancy and gestational physical health, behavioral, psychosocial, and environmental exposures in relation to birth outcomes, and whether disparities were evident in these associations according to infant sex, maternal race/ethnicity and education attainment. We identified three distinct patterns of perinatal risk via LCA, including one low risk and two differentially patterned high-risk groups, and these classes in turn yielded different associations with birth outcomes, both in the full sample and across subgroups of the population that experience health disparities. These findings are congruent with the Integrated Socio-Environmental Model of Health and Well-Being [13], demonstrating that individual, social, and environmental factors can combine and potentially interact through multifactorial pathways to influence birth outcomes.

The joint contributions of these maternal characteristics in the present study suggest that improving birth outcomes requires comprehensive interventions before or during pregnancy that consider a wide range of factors. Prior research has demonstrated the independent contribution of each perinatal risk considered in this study. For example, high BMI can lead to complications such as gestational diabetes and hypertension, which are associated with adverse birth outcome [38]. Nicotine and other harmful substances in cigarettes can restrict blood flow to the placenta, reducing oxygen and nutrient supply to the fetus [39]. Recreational drug use during pregnancy is linked to placental abruption, which increases the risk of preterm birth and low birth weight [40]. A Western dietary pattern may contribute to obesity and metabolic disorders, which correlate with preterm birth and low birth weight [41]. Poor mental health can negatively impact fetal development, as maternal stress induces hormonal changes that may hinder fetal growth [42]. Insufficient social support during pregnancy can exacerbate stress and limit access to prenatal care [43, 44]. Furthermore, heavy metals like lead and other toxicants, can cross the placenta and are linked to lower birth weight, preterm birth, and neurodevelopmental vulnerability in postnatal life [45]. Thus, the results of the present study are congruent with these independent literatures and also underscore the utility of quantifying the joint contribution and accumulated impacts of experiencing these factors simultaneously. While prior studies [38–45] have predominantly focused on isolated prenatal risk factors, the present findings suggest that co-occurring risks across physical health, behavioral, psychosocial, and environmental domains may collectively contribute to more pronounced adverse birth outcomes. This extends existing literature by demonstrating how multidomain prenatal exposures cluster together within individuals and may jointly shape fetal growth and gestational outcomes. As pregnant people and populations that experience health disparities do not experience perinatal risks in isolation, we encourage future work to examine broad sets of perinatal risks in research and practice.

Moreover, the stratified results by infant sex somewhat align with previous literature demonstrating sex-patterning in prenatal exposure and birth outcome associations [46, 47]. Male fetuses are often more adversely impacted by prenatal exposures compared to females, exhibiting worse outcomes overall [47]. Consistent with this prior research, we found that the negative prenatal effects in Class 2 were more pronounced for male infants as compared to females. For Class 3, sex-patterning was less evident. Marginal effects were observed for both males and females for birth weight, and a significant association with birth length for females but not males. Due to the lower prevalence of Class 3 in the sample, particularly in stratified models, these models may be underpowered to detect effects. We encourage future research to build on these results and further test for sex-specific associations.

When stratified by maternal race, both non-Hispanic White and racial/ethnic minority groups in Class 2 displayed similar patterns. However, racial/ethnic minority infants in Class 2 demonstrated a larger decrease in birth weight. When examining maternal educational levels, the low-education group within Class 2 exhibited increased risks for lower birth weight, reduced birth length, decreased head circumference, higher birth cephalization index, and lower gestational age at birth in weeks. Comprehensively, the results indicate the social inequalities in maternal and perinatal outcomes and align with previous research highlighting racial and educational disparities in the relationship between maternal characteristics and birth outcomes. For example, Burris and Hacker [48] reported that exposure to toxic chemicals and psychosocial stress contributes to the risk of adverse birth outcomes, with Black women often facing higher exposure levels compared to White women. Additionally, a meta-analysis by Silvestrin et al. [49] found that higher maternal education had a 33% protective effect against low birth weight. Notably, this study extends prior research by examining the joint contributions of individual, interpersonal, and environmental factors to various birth size outcomes and earlier gestational age at birth, with stratification by both maternal race and education level. The findings suggest that disparities in birth outcomes are more pronounced with respect to education than race, underscoring the importance of perinatal interventions for these populations.

This study has several limitations. First, some regression models when stratified by infant sex, maternal race/ethnicity, and education exhibited relatively poor fit, which was due in part to smaller sample sizes within strata. We chose to report results from these models as overall, they provide insights regarding disparities in infant outcomes according to maternal and infant characteristics, but we acknowledge they should be interpreted with caution. Second, although 9.4% of participants were classified into Class 3—exceeding the 1% threshold generally considered acceptable for latent classes [50–52]—future research could benefit from utilizing larger sample sizes to strengthen the robustness of the findings. Third, because LCA is a person-centered approach [18], the identified classes may not be fully generalizable to other populations. Fourth, although the latent class approach captured joint exposure patterns, potential synergistic or additive effects among exposures were not directly examined. Furthermore, the selected indicators represented only a subset of relevant maternal and prenatal exposures and should not be interpreted as a comprehensive or validated representation of all possible exposure domains. Fifth, because birth outcomes were measured once at delivery, measurement error remains possible. Finally, a potential limitation of the present study is that sex- and gestational age-adjusted standardized birth indices were not utilized for neonatal anthropometric outcomes. Although the current study focused on variation in observed birth outcomes and gestational age itself was examined as a primary outcome, future research may benefit from incorporating standardized fetal growth indicators to provide additional clinical interpretation of neonatal growth patterns.

This study also has some strengths. It employed a multimodal prospective design, integrating data from biological samples, validated questionnaires, and hospital medical records. In addition, the study’s conceptual innovation lies in simultaneously examining multiple perinatal risk factors across diverse domains, including individual, interpersonal, and environmental factors. This approach allowed for a more comprehensive understanding of how prenatal influences shape birth outcomes. Furthermore, to support this conceptual framework, the study utilized LCA, a statistical technique capable of identifying discrete groups of perinatal risk. Lastly, the study stratified the models by infant sex, maternal race/ethnicity, and maternal educational attainment, offering valuable insights into potential health disparities that may originate at birth.

## Conclusion

Understanding the influence of prenatal exposures on birth outcomes is critical, as these early-life factors can have enduring impacts on infant health and developmental trajectories. This study highlights the importance of considering a wide range of maternal characteristics when examining their contributions to birth outcomes. Additionally, researchers and clinicians can leverage these insights in designing comprehensive interventions that address multiple domains of maternal health simultaneously, particularly for closing health disparity gaps according to maternal race/ethnicity and educational attainment. By implementing strategies to mitigate perinatal risks and promoting supportive environments for expectant mothers, we can contribute to improved health outcomes for both mothers and infants, ultimately reducing health disparities that often begin at birth.

## Data Availability

The datasets used and/or analyzed during the current study are available from the authors on reasonable request.

## Abbreviations

BMI: Body mass index
LCA: Latent class analysis
AIC: Akaike information criterion
BIC: Bayesian information criterion
LMR-LRT: Lo–Mendell–Rubin adjusted likelihood ratio test
BLRT: Bootstrap likelihood ratio test
